# Peripheral eosinophilia in children with transient synovitis of the hip: 7-year experience from a single centre in New Zealand

**DOI:** 10.1007/s11832-016-0733-9

**Published:** 2016-04-15

**Authors:** Yassar Alamri, Allen Cockfield

**Affiliations:** New Zealand Brain Research Institute, 66 Stewart Street, Central Christchurch, 8011 New Zealand; Canterbury District Health Board, Christchurch, New Zealand; Department of Medicine, University of Otago, Christchurch, New Zealand

**Keywords:** Transient synovitis of the hip, Eosinophilia, Irritable hip, Limp

## Abstract

**Purpose:**

Hip pain with limping is a common presentation in childhood. The most common diagnosis in young children is transient synovitis of the hip (TSH), a benign and self-limiting condition. In our clinical practice, we observed eosinophilia in children presenting with irritable hip more commonly than would otherwise be expected. The aims of this study were to assess the prevalence of eosinophilia in children with TSH, and to evaluate the clinical outcomes of this sub-group of patients.

**Methods:**

This study retrospectively examined the data of all paediatric patients admitted to Christchurch Public Hospital, Christchurch, New Zealand. TSH cases were compared with age- and sex-matched controls.

**Results:**

A total of 103 patients were included. Compared with controls, TSH patients had significantly higher eosinophil counts (303 ± 236 vs. 380 ± 337 cells/μL, respectively, *p* = 0.049). Fourteen patients (15.6 %) had eosinophilia, with a mean eosinophil count of 986 (±321) cells/µL. Children who had eosinophilia did not differ from the rest of the sample in their age (mean 4.6 vs. 4.4 years, *p* = 0.74) or ethnicity (85.7 vs. 85.5 % European, *p* = 0.99). Eosinophilic children were not more likely to be atopic (i.e. have history of allergic rhinitis, asthma and/or eczema) than non-eosinophilic children (21.4 vs. 10.5 %, *p* = 0.37). There was a shorter hospital stay in eosinophilic children (mean 16.3 ± 6 h) than in non-eosinophilic children (mean 21.5 ± 18.8 h), although this was not statistically significant (*p* = 0.058).

**Conclusions:**

To the authors’ knowledge, this is the first study to explore the relationship between TSH and eosinophilia. We found a sizeable minority (15.6 %) of children with TSH to have eosinophilia. While the difference in hospital stay was not statistically significant, a correlation between peripheral eosinophilia and length of hospital stay of TSH patients is possible. Whether this correlation is clinically meaningful remains to be answered.

**Level of evidence:**

Retrospective prognostic study; level II.

## Introduction

Hip pain with limping is a common presentation in childhood [1]. The most common diagnosis in young children is transient synovitis of the hip (TSH), a benign and self-limiting condition that is diagnosed once other more sinister diagnoses have been excluded. TSH is sometimes confirmed by the presence of a hip effusion on radiological imaging. Whilst an exact cause is yet to be elucidated, an antecedent viral illness or trivial trauma is commonly reported [2]. Patients often present with hip pain (which may refer to the back or ipsilateral knee), decreased range of movement and/or refusal to weight-bear [2].

Epidemiological data on the incidence of TSH are generally lacking. One study from the Netherlands revealed an incidence of 76.2 cases per 100,000 person-years [1], while a recent English study found an annual incidence of 25.1 cases per 100,000 children aged 14 years or less [3]. The peak age of incidence appears to be between 4 and 10 years, with boys being more than twice as likely to develop TSH as girls [1–3].

Eosinophils represent a subgroup of granular leukocytes, whose function primarily involves combating helminthic infections and mounting eosinophil-mediated inflammatory responses [4]. Whereas eosinopaenia may be caused by either pyogenic infections or steroid excess (endogenous or exogenous), many causes have been documented for eosinophilia [5].

Eosinophilia is generally defined as an eosinophil count exceeding 450–500 cells/µL [5]. The most common cause of marked eosinophilia worldwide is infections (almost exclusively by helminths) [5]. Other causes of eosinophilia, albeit often low-grade, include medications [6], atopic and allergic diseases, malignancies (especially haematological), rheumatological diseases and the hypereosinophilic syndromes [5].

In our clinical practice, we observed eosinophilia in children presenting with irritable hip more commonly than would otherwise be expected. However, there is a paucity of published literature on the subject. Therefore, we sought to explore this observation; the aims of this study were to assess the prevalence of eosinophilia in children with TSH, and to evaluate the clinical outcomes of this sub-group of patients.

## Methods

### Study siting

This study retrospectively examined the data of all paediatric patients admitted to Christchurch Public Hospital (CPH), Christchurch, New Zealand. CPH is the largest tertiary hospital of NZ’s South Island and serves a population close to 540,000. All patients admitted between January 2009 and December 2015 were included.

The study was conducted over two stages: first, to investigate whether the observed eosinophilia was not circumstantial, eosinophil counts from identified TSH patients were compared with those of age- and sex-matched children acutely admitted for orthopaedic and trauma operations. Next, TSH patients with eosinophilia were compared to those without.

### Search strategy

Electronic records were searched using the appropriate code (M65.85) in accordance with the tenth edition of the International Classification of Diseases, Australian modification (ICD-10-AM). Identified records were searched more closely. Cases for which no diagnosis was recorded or involving patients older than 18 years or non-hip joints were excluded.

### Data collection

Admission and discharge data as well as blood results were collected from the hospital’s electronic patient record system. A re-admission was defined as an acute admission within 12 months following the admission during which TSH was diagnosed. A child was considered eosinophilic if the peak eosinophil count was higher than the age-appropriate upper normal limit. If data were not stored electronically, physical notes were sought.

### Statistical analysis

Descriptive statistics were used to analyse most of the data. Comparisons were conducted using an independent-samples Student *t* test. Statistical significance was deemed if the type I error rate was <5 % (*p*-value < 0.05). All analyses were performed using SPSS Statistics^®^ software package (version 22.0.0.0). Values are reported as mean (±standard deviation) or median (range).

## Results

### Study sample

A total of 109 patients were identified, of whom six were excluded. Three children were diagnosed with hip osteomyelitis, one child with Guillain–Barré syndrome and one child with sickle cell disease-related bone crisis. One infant was admitted for respiratory distress during which transient reduction of the right hip was observed; as there was no further evidence of TSH diagnosis, she was excluded. This left a sample of 103 patients.

### Patient demographics

The mean age on admission was 4 years (range 1–13 years). The majority of patients were male (68 %). Most patients (84.2 %) were of European origin. Right-sided TSH was slightly more common (55.8 %) than left-sided TSH. One child had simultaneous bilateral TSH and another child had no recorded side.

### Comparison with controls

All but three TSH patients had a full blood count done on admission. Of those, ten patients had a white-cell count without differential. This left 90 patients for whom eosinophil counts were available. The mean eosinophil count for our TSH sample was 380 (±337) cells/µL.

Ninety age- and sex-matched controls were included for comparison; their demographics were otherwise comparable to the TSH group. Control patients were mainly admitted for orthopaedic operations (61.8 %), appendicitis (29.4 %) and splenic injuries (8.8 %). The mean eosinophil count for controls (303 ± 236 cells/μL) was significantly lower than that of the TSH group (*p* = 0.049).

### Admission details

The most common indication for admission was to investigate the cause of a new limp in the child. The mean length of stay was slightly less than a day (20.1 ± 17.5 h). Most patients were managed by the Paediatrics team (68 %), whilst the rest were admitted under the Orthopaedics service (32 %). Ten patients (9.7 %) were re-admitted with the same presenting complaint, after a median of 3 days since initial discharge (the mean was skewed by one child who presented 79 days after initial discharge).

### Eosinophil count

Fourteen patients (15.6 %) had eosinophilia, with a mean eosinophil count of 986 (±321) cells/µL. Children who had eosinophilia did not differ from the rest of the sample in their age (mean 4.6 vs. 4.4 years, *p* = 0.74) or ethnicity (85.7 vs. 85.5 % European, *p* = 0.99). Eosinophilic children were not more likely to be atopic (i.e. have history of allergic rhinitis, asthma and/or eczema) than non-eosinophilic children (21.4 vs. 10.5 %, *p* = 0.37). There was a shorter hospital stay in eosinophilic children (mean 16.3 ± 6 h) versus non-eosinophilic children (mean 21.5 ± 18.8 h), although this was not statistically significant (*p* = 0.058).

### Management

**Fig. 1:**
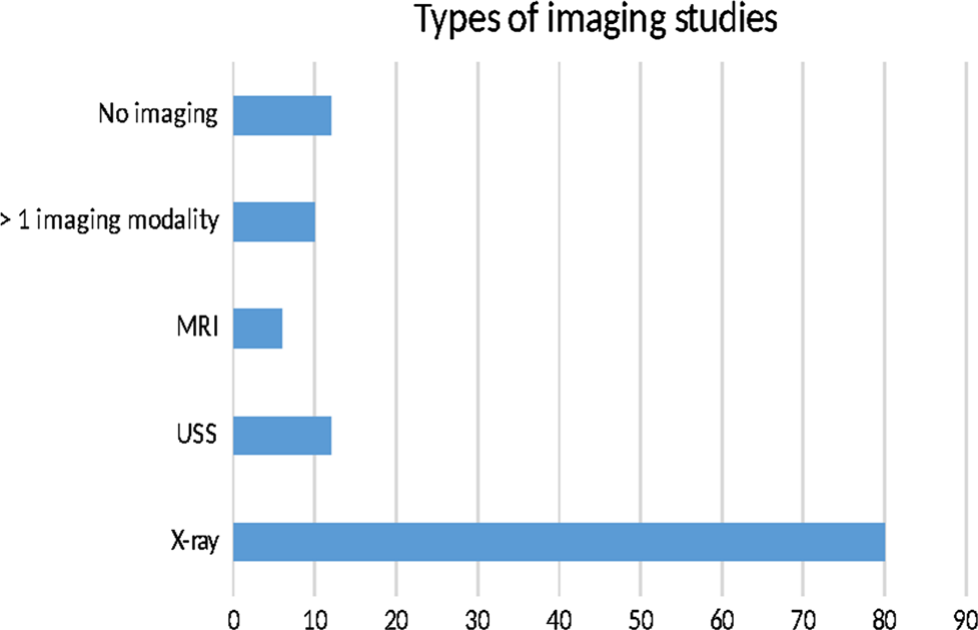
Types of radiological investigations used. Some patients had more than one modality of imaging. *MRI* magnetic resonance imaging, *USS* ultrasound scan

Most of the patients (*n* = 91) had one or more imaging studies of the affected hip on admission. The most common modality was X-ray, followed by ultrasound scan and magnetic resonance imaging (see Fig. 1). The most common abnormality was small or moderate joint effusions. There was no difference in the rate of any radiological abnormality in eosinophilic (16.7 %) versus non-eosinophilic (16.7 %) children (*p* = 1.00). None of the hip joints was aspirated. All patients were managed conservatively with observation and as-required analgesia.

## Discussion

To the authors’ knowledge, this is the first study to explore the relationship between TSH and eosinophilia. We found a sizeable minority (15.6 %) of children with TSH to have eosinophilia. This is much higher than the reported rate of 1.0–1.5 % incidental eosinophilia found in the general population [7]. To explore whether this observed eosinophilia was not circumstantial, we compared TSH children with age- and sex-matched children who had blood samples taken as part of their acute admission investigations. TSH children had significantly higher eosinophil counts than controls (380 vs. 303 cells/μL, *p* = 0.049).

TSH children in our sample did not differ in their clinical presentation or outcome from TSH patients without eosinophilia. However, they appeared to have a shorter median hospital stay (16.3 vs. 21.5 h, *p* = 0.058). While the difference in hospital stay was not statistically significant, a correlation between peripheral eosinophilia and length of hospital stay of TSH patients is possible. Whether this correlation is clinically meaningful remains to be answered.

Whilst the cause of the observed association between TSH and eosinophilia is not clearly understood, we suggest several hypotheses. A likely explanation is that a sub-group of patients with TSH, which is thought to be a reactive condition to an unknown primary trigger, may be susceptible to exhibiting eosinophilia as a reaction to the same trigger. The pathophysiology of eosinophilia in rheumatology patients is yet to be elucidated [5], although a reactive process secondary to increased circulating cytokines (especially interleukin-5) appears feasible [4].

An alternative explanation is that the observed eosinophilia may have been iatrogenic; NSAIDs (a common choice for paediatric analgesia) are a known cause of eosinophilia [6]. This hypothesis is unlikely, however, since most blood samples would have been obtained on admission prior to any medication administration. Almost 8 % of our sample (split roughly equally between TSH children with eosinophilia 3.8 %, and children without 4.2 %) were given medications by parents prior to presenting; this was, however, almost always paracetamol, a non-NSAID analgesic. Moreover, other paediatric inpatients, who are also commonly given NSAIDs, do not exhibit this eosinophilic reaction nearly as frequently as 15 %. Finally, the possibility that eosinophilic transient synovitis accounted for the observed association had to be entertained. This also is unlikely given the exceeding rarity of this clinical entity in which synovial fluid (rather than peripheral) eosinophilia is the pathological hallmark [8].

The demographics of our sample are in keeping with those reported elsewhere [1, 3]. Most of the children in our sample were between 3 and 7 years, and males were more than twice as likely as females to present with TSH. Children with eosinophilia tended, albeit non-significantly, to have shorter hospital stay. The *abnormal* result (i.e. eosinophilia) may have been an impetus for the admitting doctor to prescribe more or stronger analgesia [9, 10], leading therefore to an apparent *significant* improvement in pain and earlier discharge. This, however, remains a conjecture. In the absence of quantitative data on TSH severity or standardised pain scores, it is difficult to ascertain whether an association exists between eosinophilia and a milder course of TSH.

A few limitations inherent in the design of our study ought to be mentioned. The results presented remain a single centre’s experience evaluated in retrospect. The relationship detected between eosinophilia and TSH remains, at best, an association, since causality cannot be established with this type of study. This study, however, remains the first study to report this association. Future research should focus on larger TSH samples with longer follow-up to ascertain their disease course.

## References

[1] Krul M, van der Wouden JC, Schellevis FG, van Suijlekom-Smit LW, Koes BW (2010). Acute non-traumatic hip pathology in children: incidence and presentation in family practice. Fam Pract.

[2] Fabry G (2010). Clinical practice: the hip from birth to adolescence. Eur J Pediatr.

[3] Harrison WD, Vooght AK, Singhal R, Bruce CE, Perry DC (2014). The epidemiology of transient synovitis in Liverpool, UK. J Child Orthop.

[4] Montgomery ND, Dunphy CH, Mooberry M, Laramore A, Foster MC, Park SI, Fedoriw YD (2013). Diagnostic complexities of eosinophilia. Arch Pathol Lab Med.

[5] Curtis C, Ogbogu PU (2015). Evaluation and differential diagnosis of persistent marked eosinophilia. Immunol Allergy Clin N Am.

[6] Maidment I, Williams C (2000) Drug-induced eosinophilia. Pharm J. http://www.pharmaceutical-journal.com/learning/learning-article/drug-inducedeosinophilia/20000049.article

[7] Sims H, Erber WN (2011). Investigation of an incidental finding of eosinophilia. BMJ (Clin Res Ed).

[8] Muralidharagopalan NR, Harikrishnan V, Subbaiah S, Srinivasan C (2015). Idiopathic eosinophilic synovitis of the knee joint with peripheral eosinophilia—a rare case report. J Clin Diagn Res..

[9] Hayes J, Pehora C, Bissonnette B (2009). The use of NSAIDs in pediatric scoliosis surgery—a survey of physicians’ prescribing practice. Paediatr Anaesth.

[10] Simons J, Moseley L (2008). Post-operative pain: the impact of prescribing patterns on nurses’ administration of analgesia. Paediatr Nurs.

